# Does Flavonoid Consumption Improve Exercise Performance? Is It Related to Changes in the Immune System and Inflammatory Biomarkers? A Systematic Review of Clinical Studies since 2005

**DOI:** 10.3390/nu13041132

**Published:** 2021-03-30

**Authors:** Patricia Ruiz-Iglesias, Abril Gorgori-González, Malén Massot-Cladera, Margarida Castell, Francisco J. Pérez-Cano

**Affiliations:** 1 Secció de Fisiologia, Departament de Bioquímica i Fisiologia, Facultat de Farmàcia i Ciències de l’Alimentació, Universitat de Barcelona (UB), 08028 Barcelona, Spain; patriciaruiz@ub.edu (P.R.-I.); abrilgo2008@gmail.com (A.G.-G.); malen.massot@ub.edu (M.M.-C.); franciscoperez@ub.edu (F.J.P.-C.); 2 Institut de Recerca en Nutrició i Seguretat Alimentària (INSA-UB), Universitat de Barcelona, 08921 Santa Coloma de Gramenet, Spain; 3 Centro de Investigación Biomédica en Red de Fisiopatología de la Obesidad y la Nutrición (CIBEROBN), Instituto de Salud Carlos III, Madrid, Spain

**Keywords:** anthocyanins, cytokines, exhaustion, flavanols, inflammation, quercetin, upper respiratory tract infections

## Abstract

Flavonoids are attracting increasing attention due to their antioxidant, cardioprotective, and immunomodulatory properties. Nevertheless, little is known about their role in exercise performance in association with immune function. This systematic review firstly aimed to shed light on the ergogenic potential of flavonoids. A search strategy was run using SCOPUS database. The returned studies were screened by prespecified eligibility criteria, including intervention lasting at least one week and performance objectively quantified, among others. Fifty-one studies (54 articles) met the inclusion criteria, involving 1288 human subjects, either physically untrained or trained. Secondly, we aimed to associate these studies with the immune system status. Seventeen of the selected studies (18 articles) assessed changes in the immune system. The overall percentage of studies reporting an improved exercise performance following flavonoid supplementation was 37%, the proportion being 25% when considering quercetin, 28% for flavanol-enriched extracts, and 54% for anthocyanins-enriched extracts. From the studies reporting an enhanced performance, only two, using anthocyanin supplements, focused on the immune system and found certain anti-inflammatory effects of these flavonoids. These results suggest that flavonoids, especially anthocyanins, may exert beneficial effects for athletes’ performances, although further studies are encouraged to establish the optimal dosage and to clarify their impact on immune status.

## 1. Introduction

**Figure 1:**
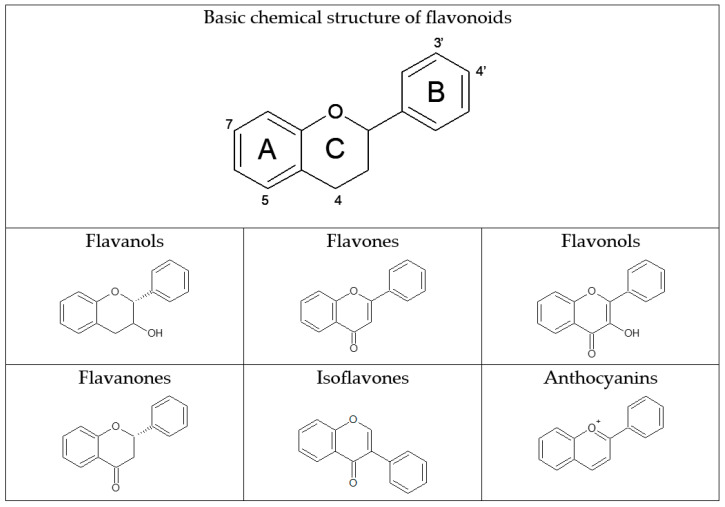
Chemical structures of flavonoids and their classes. Based on reference [1].

Among the bioactive compounds provided by diet, flavonoids are one of the most important, given that they are the most abundant polyphenols, regularly ingested in small quantities in many edible plants. Flavonoids are a broad class of secondary plant metabolites with low molecular weight and a flavan nucleus. Chemically, they are benzo-γ-pyrone derivatives consisting of a 15-carbon skeleton arranged in three rings (A, B, and C) (Figure 1). Depending on the chemical structure (hydroxylation pattern, conjugation between the aromatic rings, glycosidic moieties, and methoxy groups); degree of oxidation; and unsaturation of the linking chain, flavonoids are classified into flavanols, flavones, flavonols, flavanones, isoflavones, and anthocyanins (Figure 1) [1].

Flavonoids comprise more than 4000 compounds that are widely distributed in seeds, leaves, bark, and flowers of plants. Flavanols, as those found in green tea and cocoa, include monomers such as epicatechin, catechin, gallocatechin, epigallocatechin (EGC), and epigallocatechin gallate (EGCG) and, also, polymers called proanthocyanidins or condensed tannins. Flavones are commonly found in fruit skins, parsley, and celery and include glycosides of luteolin, chrysin, and apigenin. Flavonols can be found in onions, apples, berries, leeks, broccoli, blueberries, red wine, and tea and include, among others, quercetin, kaempferol, morin, rutin, myricetin, isorhamnetin, and isoquercetin. Flavanones are exclusive to citrus fruits and can be hesperidin, naringenin, and eriodictyol. Leguminous plants such as soy and soy products contain isoflavones such as genistein and daidzein. Finally, anthocyanins are provided by red wine and berry fruits, such as cherries, strawberries, raspberries, barberries, blueberries, and raisins, and include pelargonidin, cyanidin, and malvidin [1,2].

Flavonoid dietary intakes vary considerably among countries and cultures. It seems to be the major polyphenol class consumed by European adolescents (representing 75‒76% of the total polyphenols intake), especially the flavanol and flavanone subclasses [3]. The daily consumption of flavonoids is estimated to be 313.26 mg in Spain [4], 506 mg in France [5], 403.5–525 mg in Poland [6], and 103 mg and 80 mg in Finnish women and men, respectively [7]. Flavonoids possess antioxidant and chelating abilities. Related to these or other properties, flavonoid intakes have been associated with numerous health-promoting physiological benefits for cardiovascular disease, cancer, neurological disorders, aging, obesity, etc. [8,9,10,11,12,13,14,15]. For this reason, a wide range of human intervention studies has been developed, and the results offer promising applications in the prevention of several disorders.

With regard to sports performances, many human [16,17,18,19] and animal studies [20,21] have focused on the effect of flavonoids on several outcomes of exercise. Much of this research has studied their protective effects against the oxidative stress associated with physical activity and sports [19,22,23,24]. It is known that intense physical activity induces changes in the oxidative system of the body, leading to an overproduction of reactive oxygen species (ROS) that may disrupt the physiological balance between ROS generation and the antioxidant defense systems, producing oxidative stress [25]. Flavonoids like cocoa flavanols [19,26], green tea flavanols [27,28], and blueberry’s anthocyanins [29], among others, have demonstrated some promising success in counteracting exercise-induced oxidative stress due to their antioxidant properties.

The immune system is very sensitive to oxidative stress [30], and its function can be modulated by exercise [31]. There is a general consensus that regular bouts of moderate physical activity provide several health benefits, such as enhancing immune functionality [32,33,34]. Nevertheless, intense exercise can have detrimental effects on the immune system [32,35,36]. In general, exercise alters the phagocytic and inflammatory functions of macrophages, as well as natural killer (NK) cell functions. A moderate exercise enhances the innate immunity by increasing phagocytic and cytotoxic activities [37,38,39]. Nevertheless, intensive physical exercise has been associated with an inflammatory response [40] and a mobilization of leukocytes [41]. Eventually, there is a decrease in host defenses that leads to an increased susceptibility to infections, especially upper-respiratory tract infections (URTIs) and gastrointestinal infections, in the days following a bout of intense exercise [42].

Many studies have reported the influence of flavonoid consumption in physical activities, but when looking for the effects on performance, the number of studies decreases. Likewise, few reviews have considered physical performance as a criterion or considered only specific sports or the effects of specific supplements [17,19,43,44]. In the current article, we aimed to perform a systematic and broad (2005–2020) review based on the clinical trials (randomized controlled trials) regarding the intake of flavonoids in physical activity, looking at their influence on physical performances. We considered studies focused on healthy people aged between 18 and 50 years with flavonoid consumption, both in the pure form and as an extract, lasting for at least seven days and that objectively measured physical performances with randomized, controlled, simple, or double-blind designs. Likewise, from the studies selected, we focused on those associating changes induced by the flavonoid intake in physical performances with the immune system status.

## 2. Materials and Methods

### 2.1. Data Sources and Search Strategy

The search strategy was predetermined following the Preferred Reporting Items for Systematic Reviews and Meta-Analyses (PRISMA) guidelines [45]. The search of articles was run using the SCOPUS (Elsevier) database. The searched terms were related with flavonoids and exercise. The first searched terms were “flavonoids” AND “exercise”. Moreover, to obtain articles that may have been omitted in a general search, the concepts “athlete” OR “marathon” OR “training” OR “endurance” OR “sport” OR “players” OR “fitness” OR “cycling” were used with regards to exercise, AND “polyphenol” OR “flavanone” OR “flavone” OR “flavonol” OR “anthocyanin” OR “isoflavone” OR “catechin” OR “hesperidin” OR “glabridin” OR “quercetin” OR “blackcurrant” OR “cherry” OR “green tea extract” were searched regarding flavonoids. The strategy was first applied in July 2020 and updated on December 17, 2020 and included articles since 2005.

### 2.2. Data Selection

**Figure 2:**
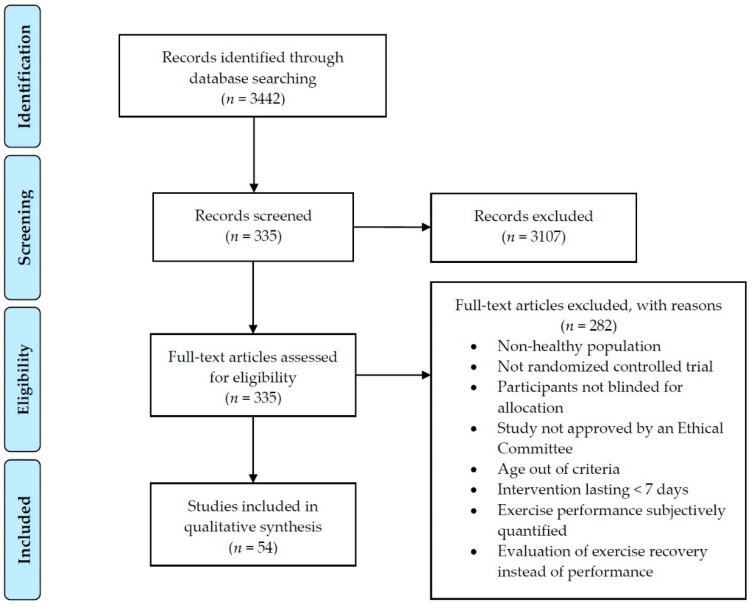
Flow diagram of the article selection process.

After running the search strategy, the inclusion and exclusion criteria were applied (Figure 2). The exclusion criteria were: (i) preclinical studies; (ii) not written in the English language; (iii) participants with morbidities (diabetes, hypertension, etc.) or overweight; (iv) conference abstracts or reviews; (v) intervention with polyphenols other than flavonoids; (vi) evaluations of only exercise recovery; and (vii) the study not approved by an Ethical committee.

The studies included in the review met the criteria: (i) healthy people aged between 18 and 50 years (mean age in the study ranging between 18 and 50 years); (ii) the study designs as randomized, controlled trial, or either single or double-blind; (iii) an intervention lasting for at least seven days; and (iv) physical exercise performances objectively quantified by means of either distance, time, work performed, anaerobic potency, anaerobic threshold, or strength.

### 2.3. Data Collection

From the selected articles after applying the exclusion and inclusion criteria, the data were collected from the entire paper. Data from (i) the study design, (ii) characteristics of the participants, (iii) flavonoid applied or its composition if there was an extract and dosage used (amount of flavonoid and length of the intervention), (iv) type of exercise, (v) performance outcomes, and (vi) the results and conclusions of the study were collected.

### 2.4. Assessment of Risk of Bias in Included Studies

The method used for assessing the risk of bias in individual studies was the Cochrane Handbook Guidelines [46]. The domains assessed were selection bias (random sequence generation and allocation concealment), performance bias (blinding of participants and personnel), detection bias (blinding of outcome assessment), attrition bias (incomplete outcome data), reporting bias (selective reporting), and other sources of bias. Each domain was categorized into “low-risk”, “high-risk”, or “unclear risk” if there was insufficient information to permit the judgment of low or high. Low-risk is interpreted as plausible bias unlikely to seriously alter the results, high-risk is interpreted as plausible bias that seriously weakens the confidence in the results, and unclear risk is interpreted as plausible bias that raises some doubts about the results.

## 3. Results

### 3.1. Study Selection

**Figure 3:**
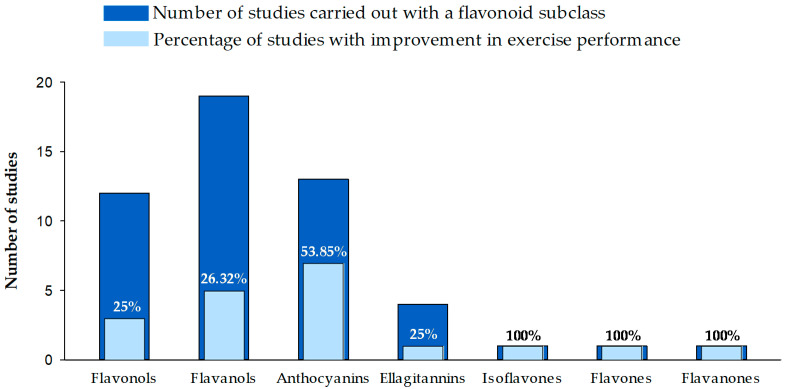
Summary of the included studies classified according to the flavonoid subclass and the percentage of studies reporting improvements in exercise performance.

A total of 3442 articles was the result of running the search strategy reported (Figure 2). These articles were screened according to the title and the abstract to apply the exclusion criteria. As a result, 335 articles could be included. However, after applying the inclusion criteria, 54 articles remained. Figure 3 summarizes the included studies classified according to the flavonoid subclass and the effect on exercise performance.

### 3.2. Study Characteristics

The studies considered were classified into two categories: those that considered a pure flavonoid (Table 1) and those that included studies carried out with extracts containing flavonoids (Table 2).

#### 3.2.1. Studies with a Single Flavonoid Supplement

From 2005 to 2020, 16 articles, referring to 14 clinical studies, were considered interventions with a single flavonoid administered in the pure form or as a combination with other compounds (Table 1). From these 16 articles [47,48,49,50,51,52,53,54,55,56,57,58,59,60,61,62], quercetin was used in 14 papers [47,48,49,50,51,52,55,56,57,58,59,60,61,62], with two articles by Nieman et al. [52,59] referring to the same clinical trial and two articles by Askari et al. [49,51] also focused on the same participants. Moreover, epicatechin [53] and hesperetin-7-O-rutinoside [54] were used in the other two studies.

- Studies with a Quercetin Supplement

Focusing on the 14 selected articles (12 clinical trials) using quercetin [47,48,49,50,51,52,55,56,57,58,59,60,61,62], all of them were randomized controlled trials and double-blinded, except for one that was single-blinded [58]. Six studies [47,48,55,56,57,58] had a crossover study design, while the six remaining [49,50,59,60,61,62] had a parallel design. The included studies involved 382 participants, of whom 335 were males. In eight of these studies [47,48,49,50,55,56,57,62], the participants’ mean age was between 19 and 23 years old, in three [58,59,61], it was between 26 and 30 years old, and, in one [60], the mean age was around 45 years old. 

Quercetin was mainly administrated in its monomeric form, and only one trial used a glycoside [56]. In 10 studies [47,48,50,55,56,57,59,60,61,62], quercetin was administrated with other compounds, such as vitamin C, tocopherols, green tea, and isoquercetin, among others. The dosage was 1000 mg per day, except in one study that used 500 mg/day [49]. The lengths of the interventions ranged between one and eight weeks. The exercise programs differed among studies: running was chosen in four studies [49,55,56,60]; cycling in four [47,48,59,63]; eccentric contractions in two [50,58]; and, in two trials, running, cycling, and strength exercises were combined [57,62]. The outcomes included distance [55], time [48,56,57,60,63], work performed [47], mean power [59], strength [50,58], or a mixture [49,62]. Some studies verified the absorption of quercetin by measuring its levels in the blood [47,52,55,56,57,59,60,62], whereas no paper reported harmful effects of this supplement. 

From the 14 articles (12 clinical trials) focused on the effects of quercetin on exercise performance, three (25%) reported beneficial effects due to the flavonoid intake, and the training used to establish this effect was cycling [48], running [55], and eccentric contractions [58]. In particular, 12 volunteers (five female and seven male) taking 1000 mg/day of quercetin dissolved in vitamin-enriched Tang for seven days underwent a substantial increase in ride time to fatigue, which was associated with a modest but significant increase in VO_2max_ [48]. Similarly, 26 physically sedentary untrained male adults were supplemented with 1000 mg/day of quercetin for two weeks and were submitted to a 60-min exercise preload and a 12-min running trial on a 15% treadmill grade with self-selected speed [55]. While no significant difference was observed in the first exercise, in the 12-min trial, the distance achieved was significantly greater during the quercetin than the placebo condition. Nevertheless, this improvement was accompanied by a trend to increase the expression of genes related to skeletal muscle mitochondrial biogenesis, which provides a partial explanation for the performance enhancement [55]. Recently, Bazzuchi et al. [58] reported that 12 young men who completed a comprehensive neuromuscular evaluation before, during, and after an eccentric protocol able to induce severe muscle damage showed a higher isometric strength in a maximal voluntary isometric contraction, as well as a lower force and muscle fiber conduction velocity decay during the eccentric exercise, after ingesting 1000 mg/day of quercetin for 14 days compared to the placebo condition. From these results, the authors suggest that quercetin, by an unknown mechanism, can attenuate the severity of muscle weakness caused by eccentric-induced myofibrillar disruption and sarcolemmal action potential propagation impairment [58].

Nevertheless, other studies involving exercise with eccentric contractions were not successful in evaluating the effect of quercetin supplements on exercise performances. O’Fallon et al. [50] analyzed the effects of quercetin supplements in an eccentric exercise and found no differences in the muscle strength and the arm angle after seven day of daily supplementation. Likewise, the evaluation of other markers altered by muscle damage, such as muscle soreness, arm swelling, and the plasma creatine kinase (CK) levels, were not modified by this flavonol.

Other studies focused on the effect of quercetin in running reported nonsignificant effects when a supplement was given for a week [56] or even for a longer period [49,51,60,62]. Abbey et al. [56] reported that the intake of quercetin-3-glucoside for seven days induced a greater percentage of fatigue decrease than the placebo in the repeated-sprint performances of team sports-trained athletes, whereas the blood interleukin (IL) 6, xanthine oxidase activity, and uric acid (related to oxidative stress) was not improved by the flavonoid supplement. The study by Sharp et al. [57] also found no difference induced by quercetin intake in eight-and-a-half days for the oxygen consumption (VO_2_ peak and respiratory exchange ratio) of physically active soldiers. Similarly, and for an even longer period, Nieman et al. [60] administered 1000 mg/day of quercetin for three weeks to 18 ultramarathoners (21 for placebo) before competing in the 160-km Western States Endurance Run and observed no significant effects due to the flavonoid supplement in the race time. Moreover, quercetin was not able to attenuate muscle soreness, inflammation, or the increase in cortisol levels, among the other biomarkers experienced by the ultramarathoners [60]. Using a longer time (eight weeks), Askari et al. [49,51] also reported no significant difference in the performances of runners or in the oxygen consumption (VO_2max_), CK, and aspartate transaminase (markers of muscle damage). Finally, in another study [62], quercetin in the form of four chews (two with breakfast and two with dinner), each one containing 250 mg of quercetin, 100 mg of isoquercetin, 100 mg of omega-3 fatty acids (eicosapentaenoic acid and docosahexaenoic acid (EPA and DHA, respectively)), 30 mg of EGCG, a vitamin mixture, sucrose, and other ingredients in a carnauba wax and soy lecithin base was administered for six weeks to 58 moderately trained men and women. Such a quercetin dosage had no significant effects in four physical performance measures (army physical fitness test, Baumgartner modified pull-up test, Wingate anaerobic test, and the 36.6-m sprint) or in the VO_2peak_.

With regard to cycling performances, although Davis et al. found significant improvements with 1000 mg/day of quercetin for seven days [48], Cureton et al., using a double-blind, pretest–post-test control group design with 30 recreationally active, but not endurance-trained, men after 1000 mg/day of quercetin supplement for 7–16 day, showed no improvements in the total work done during the 10-min maximal-effort cycling trial, the phosphocreatine recovery time constant, VO_2peak_, substrate utilization, or perception of effort during the submaximal exercise [47]. Using a longer supplementation period (three weeks), Nieman et al. [52,59], found no effects for quercetin applied to 40 cyclists after a three-day intensified exercise period (nine h of exercise) in either the changes in plasma cortisol, epinephrine, and norepinephrine levels or in immune function biomarkers, as stated in Section 3.4. On the other hand, Nieman et al. [61] also studied the effect of a two-week 1000 mg/day of quercetin, 1000 mg/day of vitamin C, 40 mg/day of niacinamide, and 800 µg/day of folic acid supplementation (1000 mg/day) and its combination with 120 mg of EGCG, 400 mg of isoquercetin, and 400 mg of polyunsaturated fatty acids (PUFAs, EPA, and DHA) on three cycling time trial performances (5-, 10-, and 20-km time trials carried out on consecutive days); mitochondrial biogenesis; immunity; or inflammation. No changes were found for the time trial finish time and cycling power output or in the mRNA expression for the gene’s peroxisome proliferator-activated receptor γ coactivator α, citrate synthase, and cytochrome c, which are related to muscle mitochondrial biogenesis. However, a two-week supplementation of quercetin and EGCG, among other nutrients, resulted in a greater granulocyte oxidative burst at the baseline and a decrease in the inflammatory and immune biomarkers, as commented on below (Section 3.4), immediately after the third exercise bout [61].

- Studies with other Pure Flavonoids

In recent years, two interesting papers that examined the effects of other pure flavonoids on the cycling performance appeared (Table 1). Schwarz el al. [53], in a double-blind, randomized, placebo-controlled parallel study, applied 200 mg/day of (–)-epicatechin to 20 recreationally active men and women in anaerobic and aerobic cycling conditions. The flavanol administration inhibited the development of a peak relative aerobic power and conferred no additional benefit for the peak anaerobic power or anaerobic capacity when compared to the placebo.

On the other hand, Overdevest et al. [54], in a randomized, parallel-group, double-blind design, administered 500 mg of citrus flavonoid extract with hesperetin-7-O-rutinoside 2S enantiomer (with a total rutinoside content of at least 90%) to 39 cyclists. They found that this flavanone increased both the absolute and relative power output in a 10-min time trial on a cycle and decreased the oxygen consumption/power ratio.

**Table 1:** Summary of the included studies assessing the effects of a single flavonoid intervention on exercise performances.

Reference	Flavonoid	Control Group	Study Design	Number of Participants (Female + Male)	Mean Age of the Participants (Years)	Dosage	Exercise	Performance Variable	Effect
**Quercetin Supplements**									
[52,59]	Quercetin + Tang powder	Tang powder	Db RPCT	0 + 40	26.1 ± 1.8 (SUP) 29.1 ± 2.4 (PL)	1000 mg/d for 3 wks	Three 3 h cycling bouts	Mean power	NS
[61]	Quercetin + isoquercetin + EGCG	Placebo +/− Quercetin	Db RPCT	7 + 32	26.3 ± 1.7 (PL) 26.8 ± 2.6 (Q) 28.1 ± 2.8 (Q + EGCG)	1000 mg quercetin + 120 mg EGCG + 400 mg/d isoquercetin for 14 d	Cycling	5, 10 and 20 km time trials	NS
[47]	Quercetin + sport hydration beverage	Sports hydration beverage	Db RPCCT	0 + 30	23.1 ± 2.4 (SUP) 22.1 ± 1.8 (PL)	1000 mg/d for 7–16 d	Cycling	Work performed in a 10 min maximal effort cycling	NS
[48]	Quercetin + Tang	Tang	Db RPCCT	5 + 7	22.9 ± 2.4	1000 mg/d for 1 wk	Cycling	Time to fatigue	Improvement
[60]	Quercetin + vit C + niacin	Placebo chews	Db RPCT	7 + 32	44.2 ± 2.0 (SUP) 46.0 ± 2.3 (PL)	1000 mg/d quercetin + 1000 mg/d vit C + 80 mg/d niacin for 3 wk	160-km Western States Endurance Run	Race time	NS
[55]	Quercetin + PowerAde Coca Cola	PowerAde Coca Cola	Db RPCCT	0 + 26	20.2 ± 0.4	1000 mg/d for 14 d	12 min running trial	Distance	Improvement
[62]	Quercetin + Isoquercetin + EGCG + Vit mix + EPA and DHA	Placebo chews	Db RPCT	14 + 44	22.0 ± 5.1 (SUP) 20.3 ± 1.6 (PL)	1000 mg/d for 6 wks	APFT, BMPU, WAnT, and 36.6 m running sprint	Time trial, repetitions, mean power and time trial	**NS**
[56]	Quercetin-3-glucoside + 6% carbohydrate sports drink	6% carbohydrate sports drink	Db RPCCT	0 + 15	23.3 ± 2.6	1000 mg/d for 1 wk	Running repeated sprints	Mean sprint time	NS
[57]	Quercetin + food bars	Energy bars	Db, RPCCT	0 + 16	22.0 ± 3.0	1000 mg/d for 8.5 d	Marching in a treadmill and cycling trial	Time trial	NS
[49,51]	Quercetin	Placebo +/- vit C	Db, RPCT	0 + 65	21.0 ± 1.6	500 mg/d for 8 wks	Running in a treadmill	Time to exhaustion or distance covered	NS
[50]	Quercetin + vit C + tocopherols	Energy bars containing vit C and tocopherols	Db, RPCT	14 + 16	19.6 ± 1.3 (female PL) 20.6 ± 1.1 (female SUP) 19.5 ± 1.1 (male PL) 20.9 ± 1.8 (male SUP)	1000 mg/d quercetin + 20 mg/d vit C + 14 mg/d tocopherols for 1 wk	Eccentric contractions of the elbow flexors	Muscle strength, arm angle	NS
[58]	Quercetin	Placebo capsules	RPCCT	0 + 12	26.1 ± 3.1	1000 mg/d for 14 d	Eccentric contractions	Arm angle, arm circumference	Improvement
**Other Flavonoid Supplements**									
[53]	(-)-epicatechin	Cellulose capsules	Db, RPCT	20	20.5 ± 1.5 (SUP) 21.0 ± 1.9 (PL)	200 mg/d for 4 wks	Cycling	Peak anaerobic power	Worsening
[54]	Hesperetin-7-O-rutinoside	Microcrystalline cellulose capsules	Db, RPCT	0 + 39	23.0 ± 0.3	500 mg/d for 4 wks	Cycling	Absolute power output	Improvement

Db = double-blind, RPCT = randomized placebo-controlled trial, RPCCT = randomized placebo-controlled crossover trial, PL= placebo, SUP = flavonoid-supplemented, d = day, wk = week, NS = nonsignificant effect, EGCG = epigallocatechin gallate, EPA = eicosapentaenoic acid, DHA = docosahexaenoic acid, APFT = Army Physical Fitness Test, BMPU = Baumgartner Modified Pull-Up Test, Vit = vitamin, and WAnT = Wingate Anaerobic Test.

#### 3.2.2. Studies with Flavonoid-Enriched Extracts

As shown in Table 2, since 2005, 38 articles have reported the effects of flavonoid-enriched extracts on sports performances [26,28,64,65,66,67,68,69,70,71,72,73,74,75,76,77,78,79,80,81,82,83,84,85,86,87,88,89,90,91,92,93,94,95,96,97,98,99], mainly using cycling and running performances but, also, strength testing, vertical jump, taekwondo, and the climbing test. These 38 articles referred to 37 clinical trials, because the articles by Nieman et al. [97] and Ahmed et al. [98] used the same clinical trial. Eighteen clinical trials applied extracts containing flavanols, 13 applied anthocyanins, 4 used ellagitannins, and flavones and isoflavones were each used in one. The length of the intervention ranged between seven days and eight weeks. From these studies, 41% (15/37) reported an improved performance after the flavonoid intake. This positive effect was obtained for 28% (5/18) studies using flavanols and about 54% (7/13) testing anthocyanins (Figure 2). The remaining improvements were observed with extracts containing ellagitannins (one out of four), isoflavones (one out of one), and flavones (one out of one).

- Studies with Flavanols

A total of 19 articles [26,28,64,65,66,67,68,69,70,71,72,74,85,94,95,96,97,98,99] referred to 18 clinical trials that applied extracts rich in flavanols as the intervention (Table 2, flavanols section). Two of these 19 articles focused on the same clinical trial [97,98]. Twelve clinical trials had a parallel design [64,65,66,67,70,71,72,94,96,97,98,99], and the six remaining had a crossover design [26,28,69,74,85,95]. Although most of the studies were double-blind, one trial was single-blind [26] and two were triple-blind [64,68]. There was a total of 495 participants involved in these studies, of whom 35 were women. Most of the studies were performed on a young population, with a mean age of between 19 and 25 years; in six studies, the participants were between 25 and 35 years [68,69,71,85,95,97,98], and in two, they were over 36 years [67,74].

The flavanol sources differed among the studies. Green tea extract was the most common [28,64,85,94,95,96,99], followed by chocolate [26,68,69,70,71], lychee [65,66], apple [74], carob [72], and three combined green tea extracts with anthocyanins [67,97,98]. The dosage and the length of the intervention also varied between studies. In nine studies, the dosage was less than 250 mg [26,65,66,67,68,69,72,85,96], three between 251 and 500 mg [64,70,71], two between 501 and 750 mg [94,95], one 751 and 999 mg [28], and four more than 1000 mg [74,97,98,99]. The lengths of the interventions ranged between one and ten weeks. The exercise protocols included running [65,66,67,71,96,97,98], cycling [26,28,68,69,74,85,94,95], strength [99], the calf-raising exercise [64], vertical jump [70], and taekwondo [72].

Flavanols were successful in enhancing performances after cycling (three out of eight reviewed studies reported ergogenic effects in cycling) [74,94,95], running (one out of six) [66], or taekwondo training (one out of one) [72]. The length of the successful interventions varied between seven days and six weeks. Ataka et al. [74] demonstrated the positive effect of the seven-day intake of Applephenon^®^, which contained procyanidins as the active components. In particular, 18 volunteers were asked to perform non-workload trials with a maximum velocity for 10 s at 30 min (30-min trial) after the start and 30 min before the end (210-min trial). The change in maximum velocities between the 30- and 210-min trials was higher in the Applephenon^®^ group than in the placebo group [74]. Similarly, Roberts et al. [94] showed the beneficial effects of a four-week administration of a decaffeinated green tea extract containing 70% EGCG (i.e., 400 mg/day) in 14 volunteers performing one hour at 50% VO_2peak_ cycling. The use of the decaffeinated green tea extract resulted in an increase in distances covered at two and four weeks of the intervention. This effect was accompanied by a higher total fat oxidation rate and a decrease in the body fat index, although the total fatty acids concentration was unaffected [94]. Similarly, Ota et al. [95] applied a green tea extract that provided 570 mg of catechins in a longer (eight weeks) study. Fourteen untrained volunteers underwent cycle exercise training twice a week during the eight-week period, and at the end, their isokinetic muscle strength, among other variables, were measured. The supplement increased their leg extension strengths without changing their muscle mass, and, in addition, there was an increase in their aerobic endurance capacities (ventilation threshold) [95].

Likewise, Kang et al. [66] demonstrated the influence of an oligomerized lychee extract (Oligonol^®^), 200 mg/day for 30 days, in a running capacity. The extract contained 15.7% flavanol monomers ((+)-catechin, and (−)-epicatechin), along with 13.3% flavanol dimers (procyanidin B2, etc.), and was able to elevate the submaximal running time and increase the anaerobic threshold when compared to the baseline values. On the other hand, Gaamouri et al. [72] applied a carob extract, which is rich in carbohydrate, dietary fiber, and polyphenols, in taekwondo athletes. In particular, carob extract is rich in flavanols such as (+)-catechin, (−)-epicatechin, (−)-epicatechingallate, EGC, EGCG, and condensed tannins and also contains considerable amounts of other polyphenols (i.e., gallic acid) [100]. After eight weeks of supplementation and training, the athletes that took the carob extract improved their distance and the maximal aerobic velocity compared to those taking the placebo after performing a final Yo-Yo intermittent recovery test level-1. The study also demonstrated that the supplement increased the weight loss of athletes [72].

Nevertheless, not all interventions with extracts containing flavanols revealed a higher performance, either applied in cycling [26,28,68,69,85,101], running [65,67,71,96,97,98] or other protocols [64,70,99]. Eichenberger et al. [85] analyzed the effects of a green tea extract (159 mg/day catechins) for three weeks in nine endurance-trained men who cycled for two h and then performed a 30-min time trial. Although no improvements in their performances, or in fat oxidation and energy expenditure, were obtained, the supplementation reduced some inflammation biomarkers, as stated below (Section 3.4.). A green tea extract in cycling performances was also applied in the study by Jówko et al. [28]. In a study including four weeks of administration of green tea extract providing 900 mg/day of catechins, the authors demonstrated that 16 sprinters who performed two repeated-cycle sprint tests did not improve their sprint performances through taking green tea extract. However, the supplement prevented the increase in blood biomarkers of oxidative stress [28].

Supplements of flavanol-enriched foods were also applied by means of dark chocolate. Allgrove et al. [26] assessed the effects of two weeks of consumption of dark chocolate in 20 active men cycling at 60% VO_2max_ for 1.5 h, with the intensity increased to 90% VO_2max_ for a 30-s period every 10 min, followed by a ride to exhaustion at 90% VO_2max_. Although dark chocolate provided 197.7 mg/day of flavanols (108.6 mg of monomeric forms and 88.8 mg of procyanidins), the time to exhaustion in the final cycling test did not differ between the supplement conditions. Nevertheless, dark chocolate consumption decreased the plasma levels of the oxidative stress biomarkers [26]. Similarly, Decroix et al. [68] studied the effects of cocoa flavanols on 14 trained cyclists in a randomized, double-blind, crossover study. The daily intake of cocoa flavanols (1765 mg/day of cocoa extract with 121 mg/day of monomeric forms) for seven days reduced the oxidative stress but did not improve the exercise performance during exhaustive exercise in hypoxia. More recently, Shaw et al. [69] also studied the effects of chocolate flavanol intake for two weeks in trained cyclists at altitude. In agreement with Decroix et al. [68], dark chocolate had no effect on the cycling performances.

With regards to flavanols in running, various studies showed no improvements in running performances. Nishizawa et al. [65] applied a lychee fruit extract (100 mg/day) containing monomers (16.3%), dimers (13.8%), trimmers (3.8%), and larger proanthocyanidins (58.6%) throughout the two-month training period of long-distance runners. The lychee fruit extract did not improve the time for a five-km track race [65]. Additionally, using long-distance runners, Scherr et al. [67] studied the effects of drinking nonalcoholic beer for three weeks, consisting predominantly of catechin, epicatechin, procyanidin B-3, and flavonols, in athletes who participated in the Munich Marathon 2009. No difference was observed in the time trial between those supplemented with flavonoids and those receiving the placebo. Nevertheless, the supplement decreased some immune outputs, as stated below (Section 3.4). The Nieman et al. group [97,98] could not demonstrate the efficacy of a mix of water-soluble polyphenols from blueberry and green tea extracts captured in a polyphenol soy protein complex (40 g/day with about 1 g/day of flavanols for two weeks) in improving the performances of long-distance runners. In addition, this supplement did not counteract the increase in inflammation (see Section 3.4) and oxidative stress biomarkers, although a distinct gut-derived phenolic signature was found [97]. On the other hand, a green tea extract combined with endurance training was tested in untrained men [96]. The supplement provided 207 mg/day of catechins and was given for four weeks, in which endurance training was performed. The flavanol-enriched extract did not improve the endurance training capacity but protected against acute exercise-induced muscle damage and oxidative stress in sedentary men [96]. More recently, García-Merino et al. [71] reported the effect of 5 g/day of cocoa (425 mg of flavanols, mainly procyanidin B2, with small amounts of flavonols, flavanones, and flavones) for 10 weeks in endurance cross-country athletes. The long intervention decreased the body and visceral fat levels of the athletes, but it did not improve their exercise performances. 

Beyer et al. [99] applied a proprietary blend of aqueous tea extracts (2 g/day for four and six weeks) from *Camellia sinensis* (green tea) containing a minimum of 40% polyphenols in untrained men and assessed their lower-body strength. *Camellia sinensis* contains EGCG, ECG, and other monomer flavanols, together with caffeine and, also, minerals such as potassium [102]. Although the four-week green tea extract supplementation increased the antioxidant capacity, six weeks of progressive resistance training showed no difference in the strengths of the supplemented, placebo, and control groups [99]. Similarly, da Silva et al. [64] evaluated the potential of green tea extract (500 mg for 15 days) on the calf-raising exercise and did not find beneficial effects from the supplement either in the number of repetitions, muscle soreness, oxidative damage, or antioxidant status. On the other hand, de Carvalho et al. [70] assessed the effect of chocolate milk with additional cocoa flavanols (308 mg) in rugby players performing vertical jumps and a Yo-Yo test to establish athlete performance. After seven days of supplementation, there were no benefits in athletic performance or in oxidative stress.

- Studies with Anthocyanins

Thirteen studies with anthocyanins were selected for the final discussion (Table 2, anthocyanins section). All of them were randomized controlled trials and double-blinded. Seven studies [73,75,76,78,79,80,83] had a crossover design, and the other six [77,82,83,84,86,87] had a parallel design. 

Around 80% of the participants were male; specifically, there were 217 males out of 263 participants. In six studies [76,78,79,81,83,84], the mean age was between 19 and 25 years old. There were three studies [77,80,82] with people aged between 26 and 30 years, one [75] between 31 and 35, and three [73,86,87] with subjects aged between 36 and 40.

In eight trials [73,75,76,77,78,79,80,81], blackcurrants was the food source of anthocyanins, and, except for one [75], it was specifically New Zealand blackcurrants (CurraNZ™). Three studies [82,83,84] were performed with Montmorency tart cherries, one [86] with purple grapes, and one [87] with blueberries. The dosage varied among studies between 105 mg/day [73,76] and 10 mL/kg/day, containing 52.6 mg/L of anthocyanins [86]. The most common dosage was between 200 and 300 mg/day [75,77,78,79,80,81,83], with only two studies using a dosage higher than 300 mg/day [87,103]. The length of the intervention in nine studies [73,76,77,78,79,80,81,82,83] was one week, with the four remaining studies being 10 days and two, three, and four weeks, respectively [75,84,86,87]. Running was the most common exercise program among the studies [75,76,77,84,86], followed by cycling [64,73,82,87] and strength through isometric exercise [78,79], intermittent forearm exercise protocol [79], and climbing [81]. Performance was measured through a time trial [73,75,77,83,84,87], maximal contractions [78,79], distance covered [76], work performed [82], and time to exhaustion [80,81,86].

Anthocyanins were able to improve the running [76,84,86], cycling [73,83], and climbing [81] performances after 7–28 days supplementation. Three studies using New Zealand blackcurrant extract (CurraNZ^®^) found a better performance after a seven days of supplementation [73,76,81]. One 300-mg capsule of CurraNZ^®^ contains 105 mg of anthocyanins—specifically, 35–50% delphinidin-3-rutinoside, 5–20% delphinidin-3-glucoside, 30–45% cyanidin-3-rutinoside, and 3–10% cyanidin-3-glucoside. Cook et al. [73] reported a 2.4% improvement in a 16.1-km cycling time trial following 30 min of steady-state cycling (3 × 10 min at 45%, 55%, and 65% VO_2max_) after seven days of intake of CurraNZ^®^ (one capsule/day). The New Zealand blackcurrant supplementation also increased the fat oxidation during cycling at 65% VO_2max_ and plasma lactate immediately after completing the time trial [73]. Perkins et al. [76] also demonstrated the beneficial effects on performance of the seven days of intake of CurraNZ^®^ (one capsule/day). Specifically, 13 active men performed a high-intensity intermittent running protocol on a treadmill, which consisted of combining 6 × 19 s of sprints and 15 s of low-intensity running. The total distance running and the distance covered during the sprints increased by 10.6% and 10.8%, respectively, after the seven-day New Zealand blackcurrant supplementation. Moreover, the post-exhaustion blood lactate levels tended to be higher after the blackcurrant intake [76]. Likewise, Potter et al. [81] showed the positive effects of seven days of CurraNZ^®^ supplementation using a higher dose (2 × 300 mg CurraNZ^®^ capsules/day, providing a total of 210 mg/day of anthocyanins) on sports climbing ability. Participants performed three climbing bouts separated by a 20-min recovery period; in each of which, they had to climb without stopping until volitional exhaustion. After supplementation, the total climbing time was increased by 23%, and the decline in climbing performance observed in the placebo condition across the repeated climbing bouts was avoided, whereas no effect was found regarding the hang time and pull-ups. No changes were observed in heart rate, blood lactate, forearm girth, or handgrip strength due to seven days of blackcurrant intake [81].

On the other hand, Braakhuis et al. [75] studied the effects of an antioxidant drink that combined blackcurrant extract and a fruit drink concentrate, providing 300 mg of anthocyanins and 15 mg of vitamin C, on training and performance in trained female runners. Participants drank 0.5 L of the antioxidant juice daily for three weeks; during which, they trained two to three times a week according to their fitness level under the supervision of one of the researchers. At the end of the supplementation period, the participants performed a 5-km time trial on a treadmill followed by an incremental running test, in which the speed and inclination were progressively increased until exhaustion. The results are quite controversial, since, whereas the fastest runners (+2 standard deviation of the mean speed on the incremental running test) showed an improved running performance in both the 5-km time trial and the incremental running test after supplementation, the average runners tended to be slower after the three weeks of intervention [75].

Despite these successful results regarding blackcurrant extract, other authors did not find changes in sports performance with a similar flavonoid source. This was the case in studies that focused on the half-marathon finish time [77], isometric maximal voluntary contractions of the quadriceps [78], and time to exhaustion during repeated intermittent forearm muscle contractions [79,80], which did not improve due to seven days of intake of 600 mg/day of New Zealand blackcurrant extract (two capsules/day of CurraNZ^®^). 

On the other hand, three articles studied the effects of anthocyanins from Montmorency tart cherries on running [84] and cycling [64,82] performances. Levers et al. [84] observed a 13% reduction in the half-marathon finish time due to a seven-day supplementation with 480 mg/day of Montmorency tart cherry powder (CherryPURE^®^, Traverse City, MI, USA), providing 991 mg of phenolic compounds and 66 mg of anthocyanins. Moreover, the tart cherry supplementation also avoided the cortisol production increase observed in the placebo condition 60 min after exercise and attenuated some of the changes induced by exercise on the muscle catabolic markers and inflammatory markers, as stated in Section 3.4 [84]. Similarly, Morgan et al. [83] observed a 4.6% decrease in the time needed to complete a 15-km cycling time trial after a 10-min steady-state cycling at 65% VO_2max_ due to a seven-day Montmorency tart cherry supplementation (CherryActive^®^ capsules, Hanworth, UK), providing 462.8 mg/day of polyphenols and 256.8 mg/day of anthocyanins. This improvement in cycling performance was accompanied by an increase in the baseline tissue oxygenation index and a higher blood lactate concentration at the end of the steady-state exercise [83]. Bell et al. [82] also studied the effect of a seven-day Montmorency tart cherry supplementation (60 mL/day of CherryActive^®^ concentrate juice, containing 9.117 mg/mL of anthocyanins) on the cycling time trial performance; however, they found no difference in the total work performed during the time trial due to the intervention. Although the supplementation did not alter the performances, Montmorency tart cherry supplementation attenuated the exercise-induced increase in lipid hydroperoxides and the inflammatory response [82].

Additionally, other anthocyanin-rich extracts have been studied in recent years. In this regard, Toscano et al. [86] showed the ergogenic effect of a 28-day integral purple grape juice intake (10 mL/kg/day containing 1.82 g/L of total phenolic compounds and 52.58 mg/L of anthocyanins) in recreational men and women runners. A 15.3% increase in the time to exhaustion was accompanied by an increased total antioxidant capacity and a higher serum content of vitamin A and uric acid, as well as a decrease in the inflammatory biomarker α-1-acid glycoprotein serum concentration [86]. On the other hand, Nieman et al. [87] analyzed the effects of a two-week freeze-dried blueberry supplementation (one cup/day blueberries equivalent, providing 345 mg of anthocyanins) and its acute combination with banana as a carbohydrate source (banana) during exercise on a 75-km cycling time trial performance and stressful exercise-induced oxylipins production. Although no significant differences in the cycling power or finish time during the time trial were found, the two weeks of blueberry intake increased the blood levels of some gut-derived phenolic metabolites [87].

- Studies with Ellagitannins

Four studies included in this systematic review used extracts enriched in ellagitannins (Table 2, ellagitannins section). All studies were double-blind, and three [88,89,90] had a crossover design, whereas one [91] was parallel. There was a total of 63 participants, and only two were women. The average ages of the participants ranged between 20 [91] and 37 [89] years old.

All studies on ellagitannins used pomegranate as the food source. The dosage was 171.9 mg/day [88], 220 and 225 mg/day [90,91], or 11.46 mg/kg/day [89]. In two of the studies, the length of the intervention was one week [88,89], whereas another lasted two weeks [90], and the fourth lasted two months [91]. Performances were measured in cycling trials through a time trial [88], average power output [89], time to exhaustion [90], or time to complete 2000 m on a rowing ergonometer [91].

Improvements in cycling performances were reported by Torregrosa-García et al. [90] in 26 amateur trained cyclists after 15 days of supplementation with POMANOX^®^ P30, providing 225 mg/day of punicalagins α and β. During the intervention period, participants had a training routine of two–four sessions per week, each session lasting at least one hour. At the end of the supplementation period, participants were submitted to an incremental exercise test to exhaustion on a cycle ergometer, in which the total time to exhaustion and time to reach ventilatory threshold 2 (previously established before starting supplementation) were greater after the 15 days of pomegranate intake. Moreover, the authors also evaluated the effects of these ellagitannins on force recovery through a repeated isokinetic unilateral leg test performed 2, 24, 48, and 72 h after inducing muscle damage through an eccentric drill protocol; however, no significant changes were found [90]. On the other hand, Trinity et al. [88] and Crum et al. [89] found no impacts of the seven-day and eight-day pomegranate ellagitannin intakes, respectively, on the cycling time trials. Finally, the Polish rowing team was used to establish the effect of a two-month pomegranate juice intake (220 mg/100 g of polyphenols) on the rowing performance, antioxidant potential, and markers of iron metabolism [91]. No differences were found in the power output and total row time over a 2000-m distance due to supplementation or on iron metabolism markers. However, they found a higher total antioxidant capacity in pomegranate-supplemented rowers one day after the 2000-m rowing test [91].

- Studies with Other Flavonoids

Two clinical trials applied other flavonoids (Table 2). One of them studied the impact of isoflavone supplementation [92]. For that purpose, 14 men daily consumed four 500-mg capsules of a proprietary blend, each one containing 150 mg of soybean peptides, 50 mg of taurine, 45 mg of *Purearia Radix* isoflavone, and 30 mg of ginseng saponin complex (STPG capsule), for 15 days. At the end of the supplementation period, the participants carried out an exhaustive cycling test at an intensity of 75% VO_2max_, where the time to exhaustion was greater in the supplemented group. This improvement in the performance after the 15-day isoflavone intervention was accompanied with higher serum concentrations of nonesterified fatty acids from 15 min of exercise onward, attenuating the decrease observed in the placebo group over the exhaustive test. Moreover, although the plasma lactate increased with exercise in both groups, lower plasma lactate levels were found after 20 and 25 min of exercise in the supplemented one. The ammonia and glycerol plasma levels also increased throughout the exhaustive test in both conditions [92].

Finally, Gelabert-Rebato et al. [93] studied the ergogenic effects of peanut husk extract (PHE) containing 95% of the flavone luteolin in combination with mango leaf extract (MLE) containing 71% of the xanthone mangiferin at low (50 mg/day of PHE and 140 mg/day of MLE) and high (100 mg/day of PHE and 420 mg/day of MLE) doses. The participants performed two exercise protocols after 48 h and 15 days of supplementation in order to assess both the acute and prolonged changes. The exercise protocols involved both low- and high-intensity stages and repeated sprinting bouts in combination with ischemia–reperfusion episodes. The 15-day intake of both tested doses of the luteolin- and mangiferin-rich extracts combination enhanced the sprint performance after ischemia–reperfusion by 22% in terms of the peak power output compared with the first exercise trial performed 48 h after starting the nutritional intervention. No changes in performances were found in the placebo group in either exercise protocols. Moreover, the supplementation with luteolin combined with mangiferin also improved the muscle O_2_ extraction and brain oxygenation [93]. 

**Table 2:** Summary of the included studies assessing the effects of flavonoid-enriched extracts on exercise performances.

Family Reference	Flavonoid source	Control Groups	Study Design	Number of Participants (Female + Male)	Mean Age of Participants (Years)	Dosage	Exercise	Performance Variable	Effect
**Flavanols**									
[74]	Apple extract (Applephenon^®^)	Crystalline cellulose capsules	Db RPCCT	9 + 9	39.1 ± 9.1	720 mg/d procyanidins for 7 d	Cycling	Change of maximum velocity	Improvement
[85]	Green tea extract	Carbohydrate-containing drink	Db RPCCT	0 + 9	32.2 ± 2.1	159 mg/d catechins for 3 wks	Cycling	Time for 30 km trial	NS
[28]	Green tea extract	Microcrystal-line cellulose capsules	Db RPCCT	0 + 16	21.6 ± 1.5	800 mg/d catechins for 4 wks	Cycling	Peak power, mean power, total work output	NS
[94]	Decaffeinated green tea extract	Corn flour capsules	Db RPCT	0 + 14	21.4 ± 0.3	400 mg/d EGCG for 4 wks	Cycling	Distance	Improvement
[95]	Green tea extract	Sports drink	Db RPCCT	0 + 14	33.9 ± 7.4	570 mg/d catechins for 8 wks	Cycling	Leg extension strength	Improvement
[96]	Green tea extract	Starch capsules	Db RPCT	0 + 40	21.0 ± 1.0	207 mg/d catechins for 4 wk	Running	Time to exhaustion	NS
[97,98]	Blueberry-green tea-polyphenol soy protein complex	Soy protein complex with non-polyphenolic food coloring	Db RPCT	13 + 18	33.7 ± 6.8 (SUP) 35.2 ± 8.7 (PL)	1001 mg/d flavanols for 17 d	Running in a treadmill for 2.5 h	Distance covered	NS
[99]	Green tea extract	Microcrystalline cellulose capsules	Db RPCT	0 + 40	23.3 ± 4.1 (CT) 21.9 ± 2.5 (SUP) 21.5 ± 2.3 (PL)	800 mg/d polyphenols for 4 wks	Maximal strength testing, lower body resistance training	Strength	NS
[64]	Green tea extract	Celulomax^®^ capsules	Tb RPCT	0 + 20	25 ± 5	18.5 mg/d catechins for 15 d	Calf-rising exercise	Number of repetitions	NS
[65]	Flavanol-rich lychee fruit extract (Oligonol ^®^)	Malt extract	Db RPCT	0 + 20	20.6 ± 1.3 (SUP) 20.6 ± 1.2 (PL)	100 mg/d flavanols for 2 months	Running training, combining low, medium, and high intensities	Time for 5-km race	NS
[66]	Oligomerized lychee fruit extract (Oligonol^®^)	Dextrin capsules	Db RPCT	0 + 38	24.6 ± 6.6 (SUP) 22.9 ± 3.6 (PL)	200 mg/d flavanols for 30 d	Running	Submaximal running time	Improvement
[67]	Nonalcoholic beer	Control beverage containing the same ingredients except for polyphenols	Db RPCT	0 + 121	44 (SUP) 42 (PL)	1.0–1.5 L/d with 47 mg/L catechin and 33 mg/L procyanidins for 3 wks	Munich marathon race	Time for the race	NS
[26]	Dark chocolate	Isocaloric control chocolate without polyphenols	Sb RPCCT	0 + 20	22.0 ± 4.0	197.4 mg of flavanols for 2 wks	Incremental cycling	Time to exhaustion	NS
[68]	Cocoa flavanols	Maltodextrin capsules containing the same amount of theobromine and caffeine than cocoa flavanols capsules	Db RPCT	0 + 14	30.7 ± 3.1	100 mg epicatechin and 23 mg catechin for 7 d	Cycling trial in normobaric hypoxia	Completed work in 20 min cycling trial	NS
[69]	Dark chocolate	Isocaloric nonchocolate placebo	Db RPCCT	2 + 10	35.0 ± 12.0	60 g/d dark chocolate for 14 d and 120 g just before trial	10 km cycling trial at altitude	Time trial	NS
[70]	Cocoa flavanols	Chocolate milk	Db RPCT	0 + 13	20.69 ± 1.49	308 mg/d flavanols for 7 d	Vertical-jump and yo-yo tests	Vertical jump performance, accumulated distance covered	NS
[71]	Cocoa flavanols	Maltodextrin	Db RPCT	0 + 32	33 ± 7 (SUP) 36 ± 8 (PL)	425 mg/d flavanols for 10 wks	Treadmill running	Time to run 1 km	NS
[72]	Carob extract	Carob- flavored commercial drink containing citric acid, sweeteners, and stabilizers	Db RPCT	11 + 12	21.91 ± 1.22	14.4 mg/d flavonoids for 6 wks	Taekwondo training + yo-yo tests	Distance covered, maximal aerobic velocity	Improvement
**Anthocyanins**									
[73]	New Zealand blackcurrant (CurraNZ™)	Microcrystal-line cellulose capsules	Db RPCCT	0 + 14	38.0 ± 13.0	105 mg/d anthocyanins for 7 d	Cycling trial	Time trial	Improvement
[75]	Blackcurrant juice	Orange flavored sports drink	Db RPCCT	23 + 0	31.0 ± 8.0	300 mg/d anthocyanins for 3 wks	Running test	Time trial	Worse for average runners, improvement for fast runners
[76]	New Zealand blackcurrant (CurraNZ™)	Microcrystal-line cellulose capsules	Db RPCCT	0 + 13	25.0 ± 4.0	105 mg/d anthocyanins for 7 d	Treadmill running	Running distance	Improvement
[77]	New Zealand blackcurrant (CurraNZ™)	Microcrystalline cellulose capsules	Db RPCT	8 + 12	30.0 ± 6.0	210 mg/d anthocyanins for 7 d	Chichester half- marathon	Finish time	NS
[78]	New Zealand blackcurrant (CurraNZ™)	Microcrystal-line cellulose capsules	Db RPCCT	0 + 13	25 ± 4	210 mg/d anthocyanins for 7 d	Submaximal isometric exercise	Isometric maximal voluntary contractions	NS
[79]	New Zealand blackcurrant (CurraNZ™)	Microcrystal-line cellulose capsules	Db RPCCT	0 + 12	25.0 ± 4.0	210 mg/d anthocyanins for 7 d	Submaximal forearm muscle contractions	Maximal volitional contraction	NS
[80]	New Zealand blackcurrant (CurraNZ™)	Microcrystal-line cellulose capsules	Db RPCCT	0 + 12	26.0 ± 5.0	210 mg/d anthocyanins for 7 d	Submaximal forearm muscle contractions	Time to exhaustion	NS
[81]	New Zealand blackcurrant (CurraNZ™)	Microcrystal-line cellulose capsules	Db RPCCT	0 + 18	24.0 ± 6.0	210 mg/d anthocyanins for 7 d	Climbing ability test	Time to exhaustion	Improvement
[82]	Montmorency tart cherry concentrate (Cherry Active^®^ concentrate juice)	Commercially cordial with less than 5% fruit, mixed with water and maltodextrin	Db RPCT	0 + 16	30.0 ± 8.0	547.02 mg/d anthocyanins for 7 d	Cycling trial	Work performed by cycling	NS
[83]	Montmorency tart cherry supplement (Cherry Active^®^)	Dextrose capsules	Db RPCCT	0 + 8	19.7 ± 1.6	256.8 mg/d anthocyanins for 7 d	Cycling time trial	Time trial completion time	Improvement
[84]	Montmorency tart cherry (Cherry PURE^®^)	Rice flour capsules	Db RPCT	9 + 18	21.8 ± 3.9	66 mg/d anthocyanins for 10 d	Running (half- marathon)	Finish time	Improvement
[86]	Integral purple grape juice	Isoenergetic carbohydrate-based beverage	Db RPCT	6 + 22	39.8 ± 8.5	10 mL/kg/d containing 52.6 mg/L anthocyanins for 28 d	Treadmill running	Time to exhaustion	Improvement
[87]	Blueberry powder	Carbohydrate and fiber-matched placebo powder	Db RPCT	0 + 59	39.0 ± 2.0	345 mg/d anthocyanins for 2 wks	Cycling	Time trial	NS
**Ellagitannins**									
[88]	Pomegranates	Carbohydrate-matched placebo drink	Db RPCCT	0 + 12	26.8 ± 5.0	171.9 mg/d ellagitannins for 7 d	Cycling in the heat	Time trial	NS
[89]	Pomegranate extract	Pure stevia extract powder	Db RPCCT	2 + 6	37 ± 11	15 mg/kg/d containing 11.46 mg/kg/d ellagitannins for 8 d	Cycling time trial	Average power outputs and energy expenditure	NS
[90]	Pomegranate extract (POMANOX^®^ P30)	Maltodextrin capsules	Db RPCCT	0 + 24	34.9 ± 10	225 mg/d punicalagins for 15 d	Cycling trial	Time to exhaustion	Improvement
[91]	Pomegranate juice (Oleofarm^®^)	Water, sugar, and grenadine	Db RPCT	0 + 19	20.8 ± 0.86(SUP) 20.9 ± 0.95(PL)	50 mL/d juice containing 220 mg/100 g polyphenols for 2 months	Rowing ergonometer	Time to complete 2000 m	NS
**Isoflavones**									
[92]	Peptides, taurine, *Pueraria* isoflavone, and ginseng saponin complex	Starch and lactose	Db RPCCT	0 + 14	21.6 ± 0.7	180 mg of isoflavone for 15 d	Cycling	Time to exhaustion	Improvement
**Flavones**									
[93]	Peanut husk extract	Microcrystal-line cellulose capsules containing maltodextrin	Db RPCCT	0 + 12	21.3 ± 2.1	50 or 100 mg/d luteolin for 15 d	Cycling trial	Peak power	Improvement

Tb = triple-blind, Db = double-blind, Sb = single-blind, RCT = randomized controlled trial, RPCT = randomized placebo-controlled trial, RPCCT = randomized placebo-controlled crossover trial. LD = low-dose, HD = high-dose, PL= placebo, SUP = flavonoid-supplemented, d = day, wk = week, and NS = nonsignificant effect.

### 3.3. Risk of Bias within Studies

**Figure 4:**
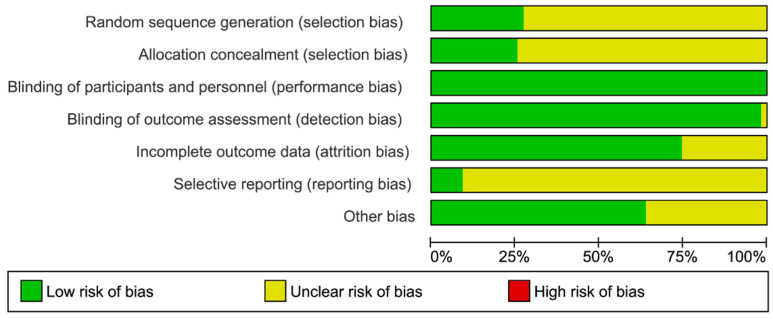
Risk of bias graph: review authors’ judgments about each risk of bias item presented as percentages across all included studies.

The risk of bias (selection bias, performance bias, detection bias, attrition bias, reporting bias, and other sources of bias) was established within the 54 articles considered (Figure 4).

In performance bias, detection bias, attrition bias, and other bias, over 50% of articles were assessed as “low-risk”. Performance bias and detection bias were assessed as “low-risk” in almost all the articles, because they assure the blinding of participants, investigators, and outcome assessment during the intervention. In the included articles, incomplete outcome data was not considered as a potential risk of bias.

The selection bias and the reporting bias scarcely went over 25%. The selection bias domain was assessed as an “unclear risk” in most of the articles, because the authors did not specify how a random sequence was generated, and, consequently, it provided an inappropriate way to evaluate the allocation concealment. In most of the studies, the reporting bias domain was assessed as an “unclear risk” due to a lack of an available protocols or not having information enough to assess this domain.

Another bias considered was if studies did not include a detailed explanation about the participation of sponsors in the intervention and the subsequent results.

### 3.4. Association between Flavonoid Intake, Performance, Immune System, and Inflammatory Biomarkers

Besides the influence of the flavonoid consumption in exercise performances, we aimed to establish the relationship of these effects with the immune system functionality of the participants. From the 54 articles selected in the systematic review, only 18 articles included measures of the immune system (Table 3). From these articles, six articles referred to the quercetin administration [50,52,56,59,60,61], with two of them focused on the same population [52,59]; six other articles applied extracts with flavanols [26,65,67,85,97,98], with two of them focused on the same clinical trial [97,98]; five articles used extracts enriched in anthocyanins [77,82,84,86,87], and one referred to a pomegranate juice with ellagitannins [91]. Most of these studies focused on the inflammatory response associated with the exercise, which was quantified by means of plasma C-reactive protein (CRP) and inflammatory and anti-inflammatory cytokines, mainly the myokine IL-6. From the 18 selected articles, only two quantified a biomarker of acquired immunity, such as salivary immunoglobulin A (IgA) [52,61]; moreover, two articles focused on the incidence of URTI [52,67], and one determined the ex vivo antibacterial and antiviral activities [98].

Firstly, with regards to those studies focused in quercetin supplementation, Nieman et al. [59] determined the levels of plasma inflammatory cytokines and chemokines such as IL-6, IL-1ra, IL-8, monocyte chemoattractant protein-1 (MCP-1), tumor necrosis factor (TNF) α, and the anti-inflammatory IL-10, as well as the leukocyte and muscle gene expression of IL-1ra, IL-8, and IL-10 in subjects submitted to three three-hour cycling bouts. The cytokine levels increased after exercise. Quercetin supplementation (1000 mg/day for three weeks) did not improve the physical performance and was only able to reduce the leukocyte gene expression of the inflammatory IL-8 chemokine and the anti-inflammatory IL-10 cytokine [59]. Nevertheless, the immune function was also studied in the same subjects by means of the NK cell activity, proliferative activity, polymorphonuclear oxidative-burst activity, and the levels of salivary IgA. None of these immune function markers were affected by the quercetin administration, but there was a lower incidence of URTI in the two-week postexercise period in supplemented cyclists compared to the placebo group [52]. In another study, Nieman et al. [60] measured inflammation by means of CRP, IL-1ra, IL-6, IL-8, IL-10, MCP-1, TNF-α, granulocyte colony-stimulating factor (G-CSF), and macrophage inflammatory protein (MIP-1β) and the leukocyte gene expression of some cytokines in ultramarathoners competing in the 160-km Western States Endurance Run who received 1000 mg/day of quercetin for three weeks before the race. In this case, quercetin was also unable to modify the physical performance or attenuate the CRP-increased levels or the increases in the plasma cytokines, and it also failed to attenuate the muscle damage. This was suggested to be due to the extreme exertion induced by running a 160-km trail race.

Nieman et al. [61] also studied the effect of two weeks of 1000 mg/day of quercetin administered with vitamin C (1000 mg/day) together or not with EGCG (120 mg), isoquercetin (400 mg), and PUFA (400 mg). As commented on above, no effects on the cycling performance by any supplement were found, but there was a greater granulocyte oxidative burst at the baseline and a decrease in plasma CRP, IL-6, and IL-10 immediately after the exercise bout [61]. The blood leukocyte count and salivary IgA were also established in these athletes. The blood leukocyte number tended to be lower after exercise in the quercetin and quercetin plus ECGC groups compared to the placebo, with significant lower levels 14 h after exercise, but no significant differences were found due to exercise or supplementation in the ratio of salivary IgA to protein [61].

On the other hand, it was reported that one week of quercetin-3-glucoside supplement was not able to prevent the increase in plasma IL-6 levels associated with repeated sprints of team sports-trained athletes or increase in their performances [56]. Similarly, quercetin did not modify the plasma IL-6 and CRP levels and did not prevent the strength loss, muscle soreness, reduced arm angle, CK elevation, and arm swelling in individuals performing two separate sessions of 24 eccentric contractions of elbow flexors [50].

Some studies focused on the extracts containing flavanols, anthocyanins, or ellagitannins, and physical performances have also shown the effect of supplements on the immune system (Table 3). A supplement of green tea extract containing 159 mg/day of catechins (flavanols) was unable to modify the cycling performance, and in comparison with the placebo group, there was also no difference in the inflammatory IL-6 cytokine, but there was a decrease in the plasma CRP levels [85]. Moreover, flavanols from a complex of blueberry–green tea–polyphenol soy protein (1001 mg/day containing 2136 mg of gallic acid equivalents for 17 days) did not improve the running distance of trained long-distance runners or prevent the biomarkers of inflammation such as the white blood cell count, plasma CRP, IL-6, and MCP-1 levels [97]. Interestingly, the immune system function of these athletes was established by means of ex vivo studies about antibacterial and antiviral activities [98]. No effect on the growth of Gram-negative and Gram-positive bacteria was found; however, the blueberry–green tea–polyphenol soy protein complex showed, by unknown mechanisms, a protective effect on virus infectivity [98]. These results suggest the potential of this flavanol mixture in the protection against viruses that often occur following intensive exercise.

Another extract enriched in flavanols, such as a lychee fruit extract with no effect on running performance in comparison with placebo group, showed interesting findings in immune-related biomarkers. Participants with supplement exhibited a lower white blood cell count increase after the training period, although no changes in neutrophil or lymphocyte counts were observed throughout the training period [65]. CRP levels and absolute serum IL-6 concentrations were not modified by the supplementation. However, the percent decrease in IL-6 from the pre-training to mid-training period was significantly smaller in the participants taking lychee extract. Similarly, the levels of anti-inflammatory cytokines such as IL-10 and transforming growth factor (TFG) β1 and β2 showed that, although the absolute concentrations were not significantly modified, the percentage increase from pre-training to post-training was significantly greater or tended to be higher for TFG-β1 and TFG-β2 concentrations, respectively, between the flavanol-supplemented and placebo groups [65]. The preventive effect of flavanols in some changes in the immune system is reinforced by the study by Scherr et al. [67]. In this case, a nonalcoholic beer providing about 47 mg/day of catechin and 33 mg/day of procyanidins for three weeks in runners of the Munich marathon prevented the increase in IL-6, CRP, and total blood leukocyte counts. Interestingly, this study demonstrated that the incidence of URTI was lower in the nonalcoholic-runner group in the two weeks after marathon competition, a period in which the supplement was also given [67]. On the other hand, Allgrove et al. [26] also assessed the effect of a two-week flavanol intake (197.7 mg/day) by means of dark chocolate in cycling. Dark chocolate did not improve the cycling performance but decreased the plasma levels of the oxidative stress biomarkers without affecting the plasma concentration of cytokines, such as IL-6, IL-10, and IL-1ra, or the blood counts of leukocytes and neutrophils after prolonged exercise [26].

As reported above, the effect of supplements containing anthocyanins has been studied in exercise performances and, in some cases, in biomarkers of the immune system. In a recent study performed in Chichester, half-marathon runners received 210 mg/day of anthocyanins from New Zealand blackcurrants; however, the flavonoids did not modify the finish time, and there was also no change in the urine IL-6 concentration [77]. Another study with anthocyanins, but from Montmorency tart cherries (547.02 mg/day anthocyanins for seven d) in well-trained cyclists, analyzed the levels of blood inflammatory cytokines and high-sensitivity CRP (hsCRP) after a stochastic road cycling trial for three consecutive days [82]. Whereas nonsignificant improvement was found for the cycling work performed, the increase in plasma IL-6 and hsCRP was attenuated by the Montmorency tart cherry concentrate, which also showed a significant effect on the oxidative stress markers. However, no influence of anthocyanins was reported for the increased levels of IL-1β, IL-8, and TNF-α [82].

Levers et al. [84] also analyzed the effect of Montmorency tart cherries but derived from tart cherry skins obtained after juicing, with a lower content of anthocyanins (66 mg/day) and for a longer period (10 days). Endurance-trained runners or triathletes racing in a half-marathon taking such a supplement produced a faster race and also experienced attenuated markers of muscle damage, oxidative stress, inflammation, and perceptions of muscle soreness than the placebo group. With regards to the inflammatory response, the serum levels of inflammatory cytokines (TNF-α, interferonγ, IL-1β, IL-2, IL-6, IL-8, and IL-12p70) and anti-inflammatory cytokines (IL-4, IL-5, IL-7, IL-10, and IL-13) were measured. The serum IL-6 concentration was attenuated by the extract in the measures 60 min post-run, whereas IL-2 and IL-13 were significantly decreased by anthocyanins at 60 min, 24 h, and 48 h of running. On the other hand, the analysis of the total and differential white blood cell counts and the granulocyte-macrophage colony-stimulating factor (GM-CSF) showed no significant changes between the placebo and supplemented groups.

Similarly to the study by Levers et al. [84], Toscano et al. [86] applied anthocyanins in recreational runners and observed an improvement in the time-to-exhaustion. In this case, the supplement derived from purple grape juice from Brazil contained 52.6 mg/L of anthocyanins and was administered at 10 mL/kg/day for 28 days. The analysis of serum α-1-acid glycoprotein (AGP) and hs-CRP concentrations and the blood total and differential white cell counts showed that the AGP levels decreased by grape juice at 14 and 28 days, with nonsignificant effects on the hs-CRP or on white cell counts. In parallel, there was an increase in the antioxidant activity by the extract [86].

More recently, as reported by Nieman et al. [87], anthocyanins from blueberries (345 mg/day for two weeks), together or not, with acute banana intake before a 75-km cycling trial did not improve the performance or plasma IL-6 and IL-1ra concentrations, although the banana consumption decreased the IL-1ra levels. This study also focused on other plasma inflammatory biomarkers such as oxylipins generated during stressful exercise from the n-6 and n-3 PUFA metabolism by the cyclooxygenase, lipoxygenase, and cytochrome P450 pathways [104]. Some of these biomarkers (those derived from the cytochrome P450 pathway) decreased due to both blueberry and/or banana ingestion.

Finally, another family of flavonoids, ellagitannins, has been studied both in exercise performance and the immune system. As reported before, Urbaniak et al. [91] used a supplement of pomegranate juice (two months) rich in ellagitannins in rowing on an ergonometer. The authors observed a higher antioxidant capacity after pomegranate fruit juice ingestion, although there was no improvement on the time to complete two km of rowing. The serum IL-6 concentration analysis also showed no changes in this inflammatory cytokine between the placebo and supplemented groups.

In summary, considering the clinical trials that applied flavonoids and quantified physical performance and immune system status, only two administering extracts with anthocyanins [84,86] showed an improvement in the exercise performance and measured some immune markers. Both these studies showed that anthocyanins lowered the inflammatory response after the quantification of IL-6 [84] or α-1-acid glycoprotein [86] but did not modify other inflammatory cytokines, CRP, or the white blood cell counts. In comparison with the other three studies using anthocyanins and determining the immune functions, the successful studies used lower the flavonoid intake (52.6–66 mg/day), but it was administered for a longer period (10–28 day) in runners, whereas the other three studies focused on cyclists or runners taking more than 200 mg/day of anthocyanins for 7–14 days (Table 3). On the other hand, extracts with flavanols [67,98] or quercetin alone [52] that did not improve the physical performance were able to decrease the incidence of URTI in athletes after intense exercise (in one case, a marathon race) or increase the ex vivo antiviral activity.

## 4. Discussion

The aim of the current review was to systematically assess the available evidence published in the last 15 years about the potential benefit of flavonoids on human sport performance when consuming them for at least seven days. To our knowledge, this is the first systematic review of the effect of flavonoids, the most consumed polyphenol class [3], on human exercise performances. From 2005 to 2020, 54 articles were selected according to the established criteria (healthy adult people, randomized, controlled trial, either single- or double-blind study designs, interventions lasting for at least seven days, and physical exercise performance objectively quantified).

The overall proportion of the reviewed articles that clearly showed an improvement in athletic performance due to flavonoid supplementation was 37% (representing the 27.5% of the participants included in this review, 30.8% women and 27.1% men), which does not allow the conclusion to be made with certainty that flavonoid consumption provides ergogenic effects. Nevertheless, when considering the different flavonoid subclasses separately, anthocyanins seem to exert a greater ergogenic effect, because the proportion of successful results after their consumption increases to 54%.

Considering a pure flavonoid intake, nearly all the studies focused on quercetin. Of these, only 25% (3/12) demonstrated that quercetin improved exercise performances [48,55,58]. Nieman et al. [55] and Davis et al. [48] hypothesized that the outcome could depend on the fitness level of the participants and suggested that the performance is more likely to be improved due to quercetin in untrained subjects, since trained participants have already reached a higher threshold regarding the antioxidant and mitochondrial capacities [105]. In fact, the three studies that confirmed quercetin’s ergogenic properties were carried out in untrained moderately active people [48,55,58], whereas the studies using trained male cyclists [52,59,63], endurance runners [60], student athletes [49,51], team sports-trained athletes [56], and military trained participants [57,62] found no beneficial effects on sports performances. However, Cureton et al. [47] and O’Fallon et al. [50] also studied the potential effects of quercetin on untrained subjects and found no changes, although the first of these authors reported a nonstatistically significant 2.7% increase in VO_2max_ following the supplementation that was not observed in the placebo condition. The observation of nonergogenic effects by these authors seems not to be related to an inadequate dose/duration of the supplementation, since Davis et al. [48] reported an improvement after just one week of 1000-mg/day quercetin intake. On the other hand, the short half-life of quercetin (3.5 h) is known [106], and in two studies reporting an improvement in performance, quercetin was consumed one h [48] and two h [59] before the exercise test, which could be important for enhancing the sports ability. Additionally, it could be hypothesized that the improvement could be due to the interaction of quercetin with other ingredients that could influence its bioavailability. Nevertheless, in the studies reporting an improvement in performance, quercetin was administered both alone [58] or in sugar-free sports beverages provided by Tang (Kraft Foods) [48] or Coca-Cola [55]. Moreover, in the unsuccessful study by Cureton et al. [47], quercetin was also consumed in a sports hydration beverage prepared by the Coca-Cola Company, and in the study by O’Fallon et al. [50], participants received quercetin through First Strike nutrition bars (Natick Soldier Center), which also contained vitamin C and tocopherols. In fact, most of the studies provided quercetin in combination with other food components, such as vitamin C, folate, PUFA such as EPA and DHA, and even other flavonoids that could influence the quercetin absorption. Although there is increasing evidence that these combinations may enhance quercetin’s bioavailability and bioactive properties, as observed in both the preclinical [107,108] and clinical studies [61,109], both successful and unsuccessful studies reported the quercetin levels in the blood [47,52,55,56,57,59,60,62]. Nevertheless, it is known that quercetin bioavailability exhibits a high interindividual variation, which can be due, among other factors, to genetic polymorphisms, dietary adaptation, the composition of gut microbiota, and other subject characteristics such as the body mass index [110]; therefore, the controversial outcomes reported could be due to intrinsic individual factors beyond its dosage.

Several mechanisms could explain the potential ergogenic effects of quercetin. In preclinical studies performed in sedentary mice, quercetin has been shown to stimulate mitochondrial biogenesis throughout enhancing the muscle and brain mRNA expression of sirtuin 1 (SIRT1) and peroxisome proliferator-activated receptor gamma coactivator 1α (PGC-1α), as well as increasing the mitochondrial DNA (mtDNA) and cytochrome C concentrations in the muscles and brain, leading to an increase in the skeletal muscle oxidative capacity and running performances [111]. In addition, an increase in mitochondrial biogenesis and muscle oxidative capacity may alter the substrate utilization during long bouts of endurance exercise by increasing the oxidation of fat and sparing muscle glucose and glycogen reserves [48]. Nevertheless, previous studies have assessed the effects of quercetin supplementation on fuel utilization in both untrained [47] and well-trained [112] cyclists, and no clear effects were found. The antioxidant properties of quercetin could also explain the potential ergogenic effect by reducing the muscle damage and soreness, as well as attenuating the decline observed in the neuromuscular performance due to the increased ROS production during exercise [58]. Moreover, in vitro studies suggest that quercetin may be an adenosine A_1_-receptor antagonist [113] and, hence, may exert analgesic effects that could decrease the effort perception or muscle aches and pain during exercise.

Regarding other pure flavonoid supplementation strategies, Overdevest et al. [54] found a 5% increase in the cycling power output after four weeks of 500 mg/day of hesperidin supplementation, a comparable improvement with that observed after creatine supplementation [114]. These positive results are even more relevant when considering that the participants were well-trained athletes with an average exercise time per week of 9.6 h. The authors suggested that this performance improvement could be due to hesperidin’s antioxidant properties, as well as other intracellular effects at the mitochondrial level, in a similar manner as previously explained for quercetin [54]. In agreement with these findings, Martínez-Noguera et al. [115] reported an increase in average power, maximum speed, and total energy during a repeated cycling sprint test only five h after the acute intake of 500 mg of 2S-Hesperidin (Cardiose^®^). Moreover, the preclinical research also supported the ergogenic effect of hesperidin [21] and its positive effects against exercise-induced oxidative stress [116,117]. Further studies should be carried out to confirm these promising results and establish the optimal intake duration for achieving these effects.

Flavanols from cocoa, administered as cocoa flavanol capsules [68], cocoa powder [71], chocolate [26,69,70], or as a capsule containing only (-)-epicatechin [53], were also assessed for improving performances. None of (-)-epicatechin [53], dark chocolate [26,69], or cocoa flavanol [70,71] consumption resulted in a better performance in recreationally active people [26,53], trained cyclists [68,69], cross-country athletes [71], and elite rugby players [70]. However, the cocoa flavanol intake may be a good strategy to counteract exercise-induced oxidative stress [26,68] and to confer some metabolic benefits [69,71]. Decroix et al. [19] reviewed the benefits of cocoa flavanol supplementation in sports and concluded that it may attenuate exercise-induced oxidative stress, improve muscular mitochondrial efficiency and VO_2max_ in untrained subjects, and positively alter fat and carbohydrate utilization during exercise without inducing changes in the exercise performance.

Regarding other flavanol-enriched extracts, eight articles assessed the impact of green tea extract supplementation on exercise performances [28,64,85,94,95,96,97,98,99], and 25% (two out of eight) found successful results in recreationally active males [94,95]. Previous studies in mice have reported an improved endurance performance following tea catechin intake [118,119], as well as an enhanced fatty acid oxidation in the liver and skeletal muscles [119]. Moreover, a synergic effect between tea catechins and other tea components such as caffeine and theanine have been reported with regards to lipid metabolism alterations [120] and could also be found in exercise-induced changes. On the other hand, the potential ergogenic effect of lychee fruit extract was studied by Kang et al. [66] and Nishizawa et al. [65]. Where a 200-mg/day intake of oligomerized lychee fruit extract for one month resulted in successfully enhancing the submaximal running time and increasing the anaerobic threshold in regularly exercising male participants [66], the intake of 100 mg/day of flavanol-rich lychee fruit extract for two months did not alter the five-km running time performances [65]. However, Nishizawa et al. [65] observed anti-inflammatory effects in young athletes after two months of 100 mg/day of lychee supplementation, as has been previously suggested in animal models [121,122]. Kang et al. [66] also studied the effects of 30 days of oligomerized lychee fruit extract supplementation with a mixture of vitamin C (800 mg) and vitamin E (320 IU) and reported an attenuation in VO_2max_. Further research may confirm these interesting findings and establish the optimal dosage of lychee fruit extract for obtaining an improvement in performances.

Another three studies assessed the impact of other flavanol-enriched extracts on exercise performances [67,72,74], and two of them reported improvements [72,74]. In particular, apple [74] and carob [72] extracts containing proanthocyanidins, among other flavonoids, successfully improved the two-hour cycling trial performance and distance covered in response to a Yo-Yo intermittent recovery test, respectively. Ataka et al. [74] also reported no impact on the two-hour cycling trial performance following a supplementation with the antioxidant ascorbic acid (1000 mg/day for eight days), suggesting that the mechanisms through which apple procyanidins intake could improve exercise performances may not be exclusively related to their antioxidant properties. These results, together with those reported by Kang et al. [66], are in line with preclinical [123,124,125] and human studies [125] suggesting that the intake of great amounts of antioxidant vitamins could attenuate or even inhibit the improvements of the endurance capacity through the blockage of exercise-induced ROS production. With regards to other tannins, four studies [88,89,90,91] assessed the potential ergogenic effects of ellagitannins from pomegranates, especially punicalagins. Positive results were only found in trained cyclists [90]. This improvement in their performances may be due to ellagitannins’ ability to enhance the blood flow and vessel diameter. Another study [126], which was excluded from the current review for not matching the intake duration inclusion criteria, reported an improvement in time to exhaustion at 90% in highly active participants following acute pomegranate extract supplementation only 30 min before exercising. However, these acute effects of pomegranates on exercise performances could be related with their high nitrate contents [127]. Overall, 28% (5/18) of the studies reviewed reported flavanols’ ergogenic effects [66,72,74,94,95], the most promising sources being apple, green tea, lychee fruit, and carob extracts. In particular, 37.5% (3/8) of the studies assessing cycling performances observed an improvement following flavanol intervention, whereas the proportion of studies finding flavanol-induced enhancement in running performances was 25% (two out of eight). These findings, together with the several health benefits that have been attributed to dietary flavanols [128,129], deserve further investigation.

Anthocyanin supplementation seems to be the most promising strategy for improving exercise performances. Thirteen of the studies reviewed assessed the effects of this flavonoid family, and 54% (7/13) found promising results for athletes. The sources of anthocyanins include blackcurrants, Montmorency tart cherries, integral purple grape juice, and blueberries.

Eight studies focused on New Zealand blackcurrant (NZBC) supplementation, containing mainly the anthocyanin delphinidin-3-rutinoside, and one-half of them reported an improvement in the performances of trained participants. It is important to highlight that these ergogenic effects were observed in either men performing the cycling exercise for 8–10 h/week [73], recreationally active men with experience in sports involving high-intensity intermittent exercise who were familiarized with treadmill running [76], and male climbers with more than three years of regular climbing experience [81]. In addition, Braakhuis et al. [75] found controversial results in trained female runners; where the average and faster (+one standard deviation of the mean speed) runners’ performances worsened following blackcurrant intakes, very fast runners (+two standard deviation of the mean speed) improved their running performances following the supplementation. They hypothesized that these results come from a synergic effect between the blackcurrant supplementation and a greater training load. In addition, Fryer et al. [79,80] found better muscle oxygenation during repeated forearm muscle contractions in intermediate, advanced, and elite climbers following NZBC supplementation that was not accompanied by an improvement in climbing performances. Nevertheless, assessing the performance changes was not the primary goal of their studies, and, as the authors stated, there were some limitations in the manner they evaluated them. Where, in advanced and elite climbers [79], the workload at 60% applied in the maximal volitional contraction (MVC) could not be intensive enough, in intermediate-level climbers [80], the coefficient of variation for the MVC was quite high (4.37%), and this could denote a lack of ability to objectively evaluate their forearm performance. Similarly, Cook et al. [78] assessed performances during submaximal isometric contractions (30% MVC), and Costello et al. [77] mainly aimed to assess changes in the recovery from half-marathon-induced muscle damage, finding no changes in the recovery of muscle function, muscle soreness, and fatigue and no differences in the half-marathon finish time.

The potential mechanisms through which blackcurrant anthocyanins could improve performances are quite unclear but seem to be related to the positive effects on endothelial function [73]. Blackcurrant anthocyanins have the potential to increase nitric oxide by endothelial cells and decrease the breakdown by nitric oxide free radicals, probably leading to enhanced skeletal muscle blood flow and contractile efficiency [73,130,131]. Overall, in agreement with Braakhuis et al., who recently reviewed the effect of NZBC on sport performances [17], we can conclude that a seven-day NZBC intake providing a daily dose of 105–210 mg/day of anthocyanins and including a final dose one to two h before exercising may result in a significant improvement in athletic performance. However, since the peak levels of anthocyanin in the bloodstream seem to appear two h post-consumption [132], further studies are recommended to elucidate whether the ergogenic effects observed reflected the seven-d intake or the acute intake.

In recent years, Montmorency tart cherries have gained increasing attention, especially with regards to their potential clinical applications in exercise-induced muscle damage recovery, inflammation, and oxidative stress [133]. From the three reviewed articles assessing their potential ergogenic effects [82,83,84], 66.6% (2/3) found successful results in endurance-trained runners or triathletes [84] and trained-male cyclists [83]. Bell’s study [82] focused on assessing the effects of Montmorency tart cherry concentrate on oxidative stress, inflammation, and muscle damage biomarkers, as well as on the expected decrease in performance across three consecutive days of cycle racing; they reported the effects on the cycle work performed as a secondary outcome, which could influence the non-observation of significant changes. As the authors suggested [82], further research should focus on functional performance assessments. The mechanisms through which tart cherries may improve exercise performances seem to be related with their high contents in cyanidin-3-glucoside. This anthocyanin has been demonstrated to enhance mitochondrial biogenesis in both mice [134] and a human hepatocyte cell line [135] through upregulating skeletal muscle PGC-1α, leading to an activation of lactate metabolism [134] and, finally, enhancing the skeletal muscle oxidative capacity and improving the endurance performance in a similar manner as previously suggested for quercetin [111]. Moreover, the upregulation of PGC-1α induced by the cyanidin-3-glucoside intake increases the expression of vascular endothelial growth factor α (VEGFα), improving the muscle blood supply and oxygen availability and, hence, explaining a potential improvement in exercise performances [134]. Overall, both blackcurrant and tart cherry extract intakes seem to be promising strategies to enhance exercise performances. However, the predominant anthocyanins in these two extracts are different, so the mechanisms underlying the potential ergogenic effects may differ and deserve further investigation.

Grape juice supplementation was also demonstrated to be effective in increasing performances in recreational runners who carried out four to five training sessions per week (78-min average training time/session) [86]. In addition, a recent study from the same authors concluded that just a single dose of grape juice two h before exercise is able to enhance runner performances [136]. Although the mechanisms underlying these effects remain unknown, the authors hypothesized that the anti-inflammatory, antioxidant [86,136,137], and vasodilator properties [138] of grape juice could lead to a better recovery between daily training sessions and, hence, an overall better exercise performance.

Apart from the most common studies on flavanols and anthocyanins, two studies reported the positive effects of a 15-day supplementation with isoflavones and flavones on cycling performances [92,93]. In particular, an encapsulated blend containing soybean peptides, taurine, *Purearia* isoflavone, and ginseng saponin increased the time to exhaustion in a cycling test, probably through enhancing the lipid utilization as an energy substrate while sparing the glycogen reserves, since increased nonesterified fatty acids blood levels during exercise were found [92]. On the other hand, a 15-day supplementation with a combination of peanut husk extract containing the flavone luteolin and mango leaf extract resulted in better sprint performances, probably through the facilitation of muscle oxygen extraction, the reduction of oxygen consumption during sprints, and an increase in the lactate blood levels [93]. It is worth noting that this study included two different doses, both showing similar effects, indicating that no dose response was achieved and suggesting that the effect of flavonoids may be limited, and no higher doses would for certain provide more successful effects.

Besides the influence of flavonoid consumption in exercise performances, we aimed to find the relationship between these effects with the immune system status of the participants. It is well-established that moderate physical activity enhances immune functions [32,33,34], but intense exercise induces inflammation, alters phagocytic and cytotoxic functions, decreases mucosal IgA, and increases the susceptibility to infections, especially URTI [40,42]. In recent years, flavonoids have shown immunomodulatory properties in both animal and human studies [139,140,141,142]. In fact, a recent review [142] demonstrated that flavonoid consumption decreases the URTI incidence by 33% compared to the control, and two studies [143,144] found significant reductions in URTI symptoms with cranberry beverages or garlic extract. Therefore, beyond the protective effects of flavonoids in the overproduction of ROS associated with intense exercise [25], flavonoids could counteract the immune changes and, eventually, protect against the increase in URTI incidences that often accompany intense physical exercise.

From the 54 selected articles considering the effect of flavonoid intake on exercise performance, only 18 articles referred to physical performances and the immune status [26,50,52,56,59,60,61,65,67,77,82,84,85,86,87,91,97,98], and only two [84,86] of these articles coincided with better performances induced by flavonoid consumption. Unfortunately, most of the 20 articles demonstrating the beneficial influence of flavonoid in exercise performances did not focus on the immune status. Both successful studies [84,86] showed that anthocyanins were able to increase the performances and, at the same time, lowered the inflammatory response by decreasing IL-6 [84] or α-1-acid glycoprotein [86], although they did not modify other inflammatory cytokines, CRP, or the white blood cell counts. Therefore, it is difficult to associate the improvement in performances by flavonoids with a biomarker of the immune system.

Regardless of their effect on exercise performances, most of the articles that considered the immune system focused on the inflammatory response, and actually, only three evaluated the immune functionality by means of the incidence of URTIs and ex vivo antiviral and antibacterial activities [52,67,98]. Interestingly, two studies demonstrated that the intake of quercetin (1 g/day for three weeks) [52] or a nonalcoholic beer enriched in catechins (for three weeks) [67] was able to decrease the incidence of URTIs in cyclists and marathon runners, respectively. Nevertheless, these studies did not find a better performance with flavonoid supplements. Therefore, it is also difficult to associate the protective effects on the immune system function from flavonoids with better performances.

Apart from these three studies that clearly demonstrate the improvement of immune functions with flavonoid intakes during exercise [52,67,98], other studies have demonstrated a certain anti-inflammatory activity from flavonoid consumption [59,65,82,85], and others failed to demonstrate the protective effects of flavonoids on inflammatory biomarkers [26,50,56,60,61,77,91,97]. It remains to be found what happened in the in vivo function of athletes or recreational subjects recruited in these studies, because the incidence of URTIs was not determined in such articles. Only Nieman et al. performed a wide study of biomarkers of the immune system, demonstrating that, with the administration of quercetin, the plasma inflammatory cytokines did not vary [59] or the NK cell activity, lymphocyte proliferative capacity, or salivary IgA [52], but there was a clear decrease in URTI incidence in these athletes [52]. Similarly, the same group demonstrated, in another clinical trial, there was no effect of the flavonol intake on the plasma inflammatory cytokines [97] but an increase in the antiviral activity, with no effect on the antibacterial potential [98]. Therefore, these studies allow the suggestion that plasma inflammatory biomarkers and even salivary IgA are not good predictors of what can eventually occur during the risk of infections after exercise bouts.

Comment must be made on the risk of bias in the studies included here. Although the overall assessment of bias of the included studies was low, it should not be forgotten that, in two domains, the number of articles with a low bias was around 25%. A more detailed description of the randomization process of some of the studies, as well as a more specific explanation of the allocation concealment, would allow a higher number of articles with a selection bias rated as low. Moreover, we would like to highlight that, in the case of some sponsored studies, no detailed description of the potential influence of the sponsors over the results were included.

Finally, although this review summarizes the current knowledge about the effects of flavonoids on the exercise performance and exercise-induced changes in immune and inflammatory biomarkers, some limitations need to be considered. Whereas some of the reviewed studies focused exclusively on exercise performance assessment, others reported it as a secondary outcome. For this reason, the performance assessment was established by different variables. It would be interesting if articles provided the same performance measures for a better evaluation of the flavonoid ergogenic effect—for instance, in running and cycling, the time to cover a certain distance would be very objective and easy to compare between different studies. Moreover, the included interventions were very heterogeneous; depending on the study, the flavonoid supplementations were provided in everyday foods or in dietary supplements that could influence the flavonoid absorption. In addition, the intrinsic individual factors, such as human subjects’ genetics and gut microbiota composition and functionality [145], are recognized to affect the flavonoid absorption and, eventually, influence its body effects. The doses also varied between the studies and flavonoid subclass, the average being about 430 mg/day, which could be achieved in a natural way by fruit intake. In addition, it should be noted that most of the studies were performed with recreationally active participants, trained people or even elite athletes, and the outcome may depend on the fitness level of the participants and the kind of exercise performed.

## 5. Conclusions

Although promising results have been found regarding flavonoid supplementation in sports performances, no clear conclusions can be drawn. The intake of anthocyanin-enriched extracts seems to be the most promising strategy for both enhancing physical performances and counteracting the increase in inflammation induced by intensive exercise, although further studies are encouraged to confirm these effects, establish the optimal dosage, elucidate the dose-response effect, and ascertain their impact on the immune status. 

## Figures and Tables

**Table 3:** Summary of the included studies assessing the association between flavonoid intake, exercise performance, and immune status biomarkers in humans.

Reference	Flavonoid	Dosage	Exercise	Effect on Performance	Measurement	Outcome
**Quercetin**						
[59]	quercetin + Tang powder	1000 mg/d for 3 wks	Three 3-h cycling bouts	NS	plasma inflammatory cytokines (IL-6, IL-10, IL-1ra, IL-8, MCP-1, TNF-α) leukocyte mRNA muscle mRNA	= plasma inflammatory cytokines ↓ leukocyte gene expression of IL-8 and IL-10
[52]	quercetin + Tang powder	1000 mg/d for 3 wks	Three 3-h cycling bouts	NS	NK cell activity proliferative activity PMN oxidative-burst activity salivary IgA incidence of URTI	= NK cell activity = proliferative activity = PMN oxidative-burst activity = IgA ↓ incidence of URTI
[60]	quercetin + vit C + niacin	1000 mg/d quercetin + 1000 mg/d vit C + 80 mg/d niacin for 3 wks	160-km Western States Endurance Run	NS	CRP Plasma inflammatory cytokines (IL-1Ra, IL-6, IL-8, IL-10, G-CSF, MCP-1, MIP-1β, TNF-α, MIF-1) leukocyte gene expression of some cytokines	= CRP = plasma inflammatory cytokines = leukocyte gene expression
[56]	quercetin-3- glucoside + 6% carbohydrate sports drink	1000 mg/d for 1 wk	Running repeated sprints	NS	plasma IL-6	= plasma IL-6
[61]	Quercetin + isoquercetin + EGCG	1000 mg quercetin + 120 mg EGCG + 400 mg/d isoquercetin for 14 d	Cycling	NS	plasma CRP plasma IL-6 and IL-10 blood leukocyte counts salivary IgA	= plasma CRP = plasma IL-6 and IL-10 ↓ blood leukocyte counts = salivary IgA
[50]	Quercetin + vit C + tocopherols	1000 mg/d quercetin + 20 mg/d vit C + 14 mg/d tocopherols for 1 wk	Eccentric contractions of the elbow flexors	NS	plasma IL-6 plasma CRP	= IL-6 = CRP
**Extracts with flavanols**						
[85]	Green tea extract	159 mg/d catechins for 3 wks	Cycling	NS	plasma IL-6 plasma CRP	= IL-6 ↓ CRP
[97]	Blueberry–green tea–polyphenol soy protein complex	1001 mg/d flavanols for 17 d	Running in a treadmill for 2.5 h	NS	WBC count serum CRP plasma IL-6, MCP-1	= WBC = CRP = plasma IL-6, MCP-1
[98]	Blueberry–green tea–polyphenol soy protein complex	1001 mg/d flavanols for 17 d	Running in a treadmill for 2.5 h	NS	ex vivo antibacterial activity ex vivo antiviral activity	= ex vivo antibacterial activity ↑ ex vivo antiviral activity
[65]	Flavanol-rich lychee fruit extract	100 mg/d flavanols for 2 months	Running training, combining low, medium, and high intensities	NS	Total and differential WBC counts CRP serum inflammatory (IL-6) and anti-inflammatory cytokines (IL-10, TFG-β1, TFG-β2)	↓ WBC counts, = neutrophil and lymphocyte counts = CRP = absolute IL-6, IL-10, TFG-β1, TFG-β2 ↓ % IL-6 and TFG-β1 from pre-training to mid-training period
[67]	Nonalcoholic beer	1.0–1.5 L/d with 47 mg/L catechin and 33 mg/L procyanidins for 3 wks	Munich marathon race	NS	IL-6 CRP total blood leukocyte counts incidence of URTI	↓ IL-6 ↓ CRP ↓ WBC ↓ URTI incidence
[26]	Dark chocolate	197.4 mg flavanols for 2 wks	Incremental cycling	NS	inflammatory and anti- inflammatory cytokines WBC, neutrophil counts	= IL-6, IL-1ra, IL-10 = WBC, neutrophil counts
**Extracts with anthocyanins**						
[77]	New Zealand blackcurrant	210 mg/d anthocyanins for 7 d	Chichester half-marathon	NS	urine IL-6	= urine IL-6
[82]	Montmorency tart cherry concentrate	547.02 mg/d anthocyanins for 7 d	Cycling trial	NS	hs-CRP blood inflammatory cytokines	↓ hs-CRP ↓ IL-6 = IL-1β, IL-8, TNFα
[84]	Montmorency tart cherry	66 mg/d anthocyanins for 10 d	Running (half-marathon)	Improvement	serum inflammatory cytokines (TNF α, IFN-γ, IL-1β, IL-2, IL-6, IL-8, IL-12p70) serum anti-inflammatory cytokines (IL-4, IL-5, IL-7, IL-10, IL-13) total and differential WBC GM-CSF	↓ IL-6, ↓ IL-2, = remaining cytokines ↓ IL-13, = remaining cytokines = WBC = GM-CSF.
[86]	Integral purple grape juice	10 mL/kg/d containing 52.6 mg/L anthocyanins for 28 d	Treadmill running	Improvement	serum AGP hs-CRP WBC	↓ AGP = hs-CRP = WBC
[87]	Blueberry powder	345 mg/d anthocyanins for 2 wks	Cycling	NS	Inflammatory cytokines Oxylipins	= IL-6, IL-1ra ↓ some oxylipins
**Extracts with ellagitannins**						
[91]	Pomegranate juice	50 mL/d juice containing 220 mg/100 g polyphenols for 2 months	Rowing ergonometer	NS	Serum inflammatory cytokines	= IL-6, IL-1ra

↓ = decrease, ↑ = increase, AGP = α-1-acid glycoprotein, CRP = plasma C-reactive protein, GM-CSF = granulocyte colony-stimulating factor, hs= high-sensitivity, IFN = interferon, IgA = immunoglobulin A, IL = interleukin, MCP-1 = monocyte chemoattractant protein-1, NK = natural killer, PMN = polymorphonuclear leukocytes, TGF = transforming growth factor, TNF = tumor necrosis factor, URTI = upper-respiratory tract infections, vit = vitamin, WBC = white blood cells.

## Data Availability

Data sharing not applicable.
